# Effects of Temperature on Chronic Trapezius Myofascial Pain Syndrome during Dry Needling Therapy

**DOI:** 10.1155/2014/638268

**Published:** 2014-10-14

**Authors:** Gang Wang, Qian Gao, Jingshan Hou, Jun Li

**Affiliations:** Department of Rehabilitation Medicine, The Chinese PLA General Hospital, Beijing 100853, China

## Abstract

The purpose of this study was to investigate the effects of temperature on chronic trapezius myofascial pain syndrome during dry needling therapy. Sixty patients were randomized into two groups of dry needling (DN) alone (group A) and DN combined with heat therapy group (group B). Each patient was treated once and the therapeutic effect was assessed by the visual analogue scale (VAS), pressure pain threshold (PPT), and the 36-item short form health survey (SF-36) at seven days, one month, and three months after treatment. Evaluation based on VAS and PPT showed that the pain of patients in groups A and B was significantly (*P* < 0.05) relieved at seven days, one month, and three months after treatment Compared to before treatment. There was significantly (*P* < 0.05) less pain in group B than group A at one and three months after treatment. The SF-36 evaluation demonstrated that the physical condition of patients in both groups showed significant (*P* < 0.05) improvement at one month and three months after treatment than before treatment. Our study suggests that both DN and DN heating therapy were effective in the treatment of trapezius MPS, and that DN heating therapy had better long-term effects than DN therapy.

## 1. Introduction

Myofascial pain syndrome (MPS) is a common clinical disease defined as a regional pain syndrome with characteristics of muscle pain caused by myofascial trigger points (MTrPs) [1]. MTrPs were first proposed and defined by Travell and Simons. An active MTrPs usually produces a referred pain pattern typical of that muscle, restricted range of motion and a visible or palpable local twitch response (LTR) during mechanical stimulation of the MTrPs [2]. In most cases, due to a stiff trapezius muscle, neck and upper back pain is the most common complaint in MPS [3].

Epidemiological investigations revealed that 30%–85% of patients with pain in the United States have MTrPs [4–6]. Among 96 patients with muscle pain who met with Gerwin [7], 74% is caused by MTrPs. There are many treatments of MPS and dry needing (DN) therapy is increasingly used. DN is an aggressive therapy that treats lesions with dry needling alone (such as acupuncturing MTrPs), without medication. Most researchers believe that the application of DN could be traced to 1979; when Lewit published his article, the study divided the traditional drug injection treatment into two parts examining individual treatment effects, comparing the effects from drug injection and the effects from acupuncture [8]. Since then, studies on the effect of simple acupuncture have increased and are called DN in order to distinguish it from the traditional drug injection.

Among the patients in our outpatient clinic, those with MPS accounted for a high proportion. These patients were treated by acupuncturing MTrPs using a special kind of needle with a diameter of 0.9 mm that has been used for many years in our clinic. In addition, the needle tail was heated so as to increase the temperature at the needle tip based on thermal conductivity. To our knowledge, studies on effect of needle body heat in the DN treatment are not reported yet.

The purpose of this study is to investigate if the needle tail heating will improve the effect of DN in treating chronic MPS.

## 2. Patients and Methods

### 2.1. Patients

Patients were enrolled in Medicine Center, People's Liberation Army General Hospital, Beijing, China, from April to October 2013. The inclusion criteria included patients aged 20–70 years, male or female, presenting with more than three months of trapezius MPS and with moderate to severe pain with a VAS score greater than four (0 = no pain, 10 points = worst pain). The patients had normal cognitive function, consented to participate in this study, and signed the informed consent form. The main diagnostic criteria for MPS [9] are (1) localized spontaneous pain; (2) spontaneous pain or altered sensations in expected referred pain area for given trigger point; (3) taut, palpable band in accessible muscle; (4) exquisite, localized tenderness in precise point along taut band; (5) some measurable degree of reduced range of movement. The secondary diagnostic criteria for MPS [9] are (1) reproduction of spontaneously perceived pain and altered sensations by pressure on trigger point, (2) elicitation of a local twitch response of muscular fibers by transverse “snapping” palpation or by needle insertion into trigger point, and (3) pain relief obtained by muscle stretching or injection of trigger point (Table 1).

Exclusion criteria included shoulder and neck skin disease, a history of neck surgery, use of anticoagulants, taking antiplatelet drugs within three days before the study, a history of cancer-related pain within six months before the study, receiving injections in the spot to be punctured in this study within three months before the study, receiving other treatments for MPS except for oral medications, having fibromyalgia syndrome, cervical nerve root or spinal cord lesions, a medical history of neck/shoulder operations, psychiatric or psychological disease, cancer, rheumatoid arthritis, endocrine diseases, pregnancy, severe depression or schizophrenia, obesity, and body mass index > 28 (Table 1).

### 2.2. Informed Consent and Ethical Approval

Before the study, each patient meeting the inclusion criteria was provided with an informed consent form, explained about the basic procedure to be performed for treatment and evaluation and potential risk, and informed of their rights to stop treatments at any time without undertaking any consequence. Those who agreed to participate in the treatment and subsequent follow-up were required to sign the informed consent form for the study.

### 2.3. Sample Size

We refer to the previous studies to determine the sample size [10], and the sample size was initially 23 for each group here. Taking into account the possible sample loss, we determined the sample size as 30 for each group. Hence, a total of 60 patients participated in this study.

### 2.4. Randomly Grouping

The patients with chronic trapezius MPS enrolled in this study were randomized into two treatment groups: DN at room temperature (group A) and DN combined with heat therapy (group B). The short- and long-term treatment effects of these two methods in the patients were compared.

### 2.5. Single-Blind Study

Since the patients may feel the treatment difference, the single-blind study was performed following the procedure of a double-blind study in order to minimize the effects of personal emotions of patients on the treatment. Evaluations were conducted before and after treatment, and follow-up study was conducted by specialized physician who knew nothing about the treatment. Evaluation is completed by two physicians: both of them have received specialized training. After the diagnosis, the physician confirmed that patients met with inclusion criteria but without exclusion criteria and informed the patients of acupuncture therapy randomly assigned with DN for some and DN with heating for the others. If the patients agreed to participate in the study, the physician filled out the clinical information form, sealed, and stored it. The patients were given a card with their sequence number. On treatment, the card was collected by a triage nurse. The nurse identified the treatment group of the patients in the randomization table and told the physician. The physician could introduce the treatment procedure and precautions to patients before DN but could not mention about whether heating was applied. Data were analyzed by personnel who did not participate in the grouping and treatment.

### 2.6. Tool Used for the DN Treatment

The needles (Shuxin Scientific and Technology Development Co., Ltd., Shanghai, China) were made of 85% silver and smaller amounts of metals such as copper, chromium, and nickel through smelting and die-cast process. The needle handle was five cm in length. The needle body was eight cm in length and 0.9 mm in diameter. It was strong and flexible. The needle tip was slightly blunt.

Silver needle-conducting temperature-adjusting travel-inspecting equipment, YRX-1 thermal temperature logging device (Shuxin Scientific and Technology Development Co., Ltd., Shanghai, China), was used as the heating instrument. It had 20 bamboo-style heating tubes placed around the needle handle for resistance wire heating. It was attached with temperature feedback loop with the highest temperature of 120°C and temperature fluctuation less than ±2°C around the target temperature.

Digital thermometer (Nanjing University of Science & Technology, Nanjing, China) was used. It was four-channel with a measurement range of 15–99°C, accuracy of 0.5°C, and the reaction time of 0.85 s. It included the needle semiconductor thermistor sensor with temperature needle length of 12 cm and diameter of 1 mm.

Pressure pain threshold analyzer was the SLY-HFM hand type pain threshold detector (Beijing Shuolin Scientific and Technology Development Co., Ltd., Beijing, China) with a measurement range of 0–1000 g, an accuracy of 2.5 g, and a precision of 0.1 g.

### 2.7. Procedure

**Figure 1:**
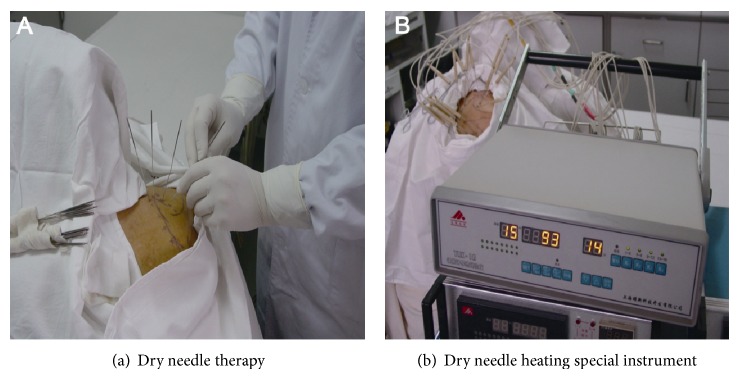
Myofascial pain syndrome treated with dry needle therapy.

**Figure 2:**
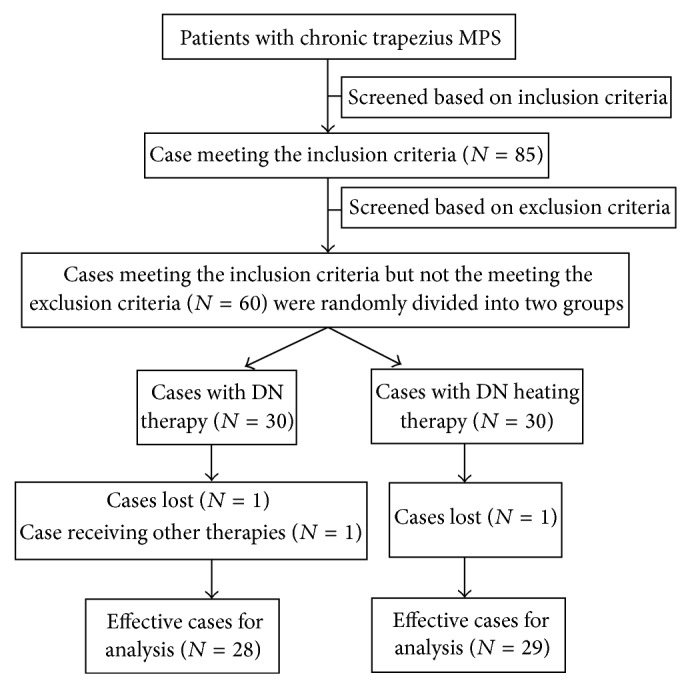
A heating instrument for dry needling therapy.

Patients in group A were treated with DN in a diameter of 0.9 mm while those in group B were treated with DN plus additional 15 minutes of heat treatment. Specifically, patients in group A lied in bed in a prone position. The spot of trapezius MTrP was determined and marked by an experienced and licensed physician. After sterilization with disinfectant and draping were completed, the doctor wore sterile gloves to identify the MTrP using the left hand and intradermally injected 0.25% lidocaine at each point. When a local hillock in diameter of about 0.8 mm was formed, the needle in the right hand was injected vertically into the skin to reach the MTrP (the correct position was assumed as long as the usual symptoms of pain or local twitch response were observed [11]). The treatment above was performed on each MTrP for 15 min. Patients in group B experienced the acupuncture following the procedure same as above, but the needles were heated using a special heating device by covering bamboo-style heating tubes over each needle tail. The thermodetector was applied to measure and adjust the temperature so as to ensure the injection temperature at 44°C. After 15 min of acupuncture, the needle was removed. Because the type of needle used was thick, a small amount of blood may ooze upon withdrawal. In order to reduce bleeding and to prevent infection, sterile gauze was used for local oppression for three min after needle removal. Then, sterile gauze was replaced to cover the treated area and fixed with adhesive tape. The patients were told to avoid contact with water in the treatment spots for 48 hours. After that, the tape could be removed. No further medication or physical therapy was performed (Figures 1 and 2).

### 2.8. Evaluation Method

The effects of DN on MPS were evaluated according to the visual analog scale (VAS) and short-form 36-item questionnaire (SF-36) [10, 12]. The pressure pain threshold (PPT) was also considered because the VAS and SF-36 methods could be affected by subjective factors.

The PPT was determined by a handheld PPT detector as followed. The detector is composed of a pressure rod about one cm^2^ attached with the rubber pad at one end, which was connected with a circular pressure gauge at the other end through the connecting rod with built-in piston. The gauge had the subgrids with 100 g for each subgrid in units of kg/cm^2^, a measurement range of 1–10 kg/cm^2^. For use, the end with the rubber pad was directed at the measurement point. The long axis of PPT was perpendicular to the skin surface over the measurement point. Then, the pressure was increased with a rate of 30 kPa/s [13]. The pressurization stopped when the patients started to feel and complained about the pain. The pressure bar was removed way from the measurement point. At this moment, the value displayed on the gauge was PPT of the measurement point. When the measurement was finished, the gauge was set to zero by pressing the zero button and pointing the pointer to its initial position, making it ready for the next measurement. The point for treatment and used as control was individually measured. During the measurement, each point was detected for three times with a measurement interval of 15 s. The average of three measurements was recorded for each point.

### 2.9. Statistical Analysis

Data were analyzed using the Statistical Package for Social Science version 13 (SPSS 13). The Chi-square test was performed for comparison of gender, age, disease duration, and whether the trigger points were located in the dominant side in patients among the two groups. Analysis of variance was used to compare the difference before and after treatments in each group. The *t*-test was for intergroup comparison at the same treatment time. A probability smaller than 0.05 (*P* < 0.05) was considered to be statistically significant. The treatment effects in patients were evaluated at 0 days (before treatment), seven days (after treatment), one month (after treatment), and three months (after treatment).

## 3. Results

VAS analogue scale showed that the pain in patients of group A was relieved after seven days, one month, and three months after treatment than before treatment. But there were not significantly different of pain in seven days, one month, and three months after treatment. Similarly, the pain in patients of group B was also relieved after seven days, one month, and three months after treatment than before treatment. And there were not significantly different of pain in seven days, one month, and three months after treatment (Table 2).

**Figure 3:**
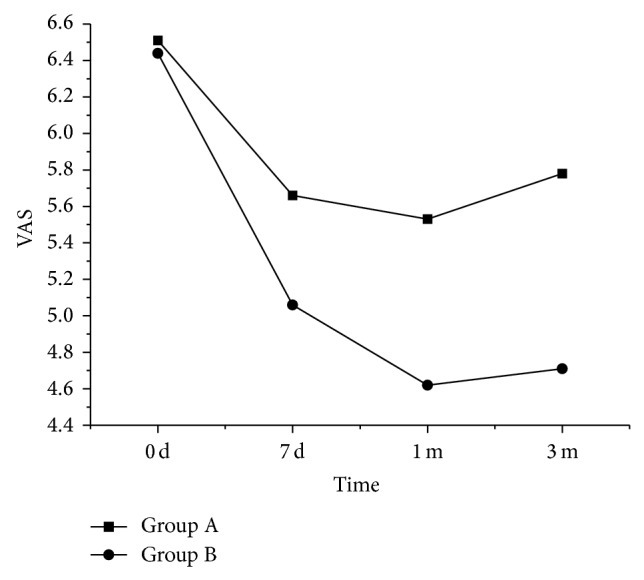
VAS analogue scales before and after treatment in patients of groups A and B.

The degree of pain in patients was similar between groups A and B before treatment and in seven days of treatment. However, the pain in patients of group B was lighter than that in group A in one and three months of treatment (Figure 3).

The SF-36-based evaluation demonstrated that, compared with before treatment, the quality of life of patients in group A was not improved after seven days of treatment but greatly improved in one and three months after treatment. There was no significant difference in quality of life of patients of group A between seven days of treatment and one month of treatment and between one and three months of treatment. However, the quality of life of patients in group A was better in three months of treatment than in seven days of treatment. Compared with before treatment, the quality of life of patients in group B was not improved after seven days of treatment but greatly improved in one and three months after treatment. There was no significant difference in quality of life of patients of group B between one and three months of treatment. However, the quality of life of patients in group B was better in one and three months of treatment than in seven days of treatment (Table 3).

**Figure 4:**
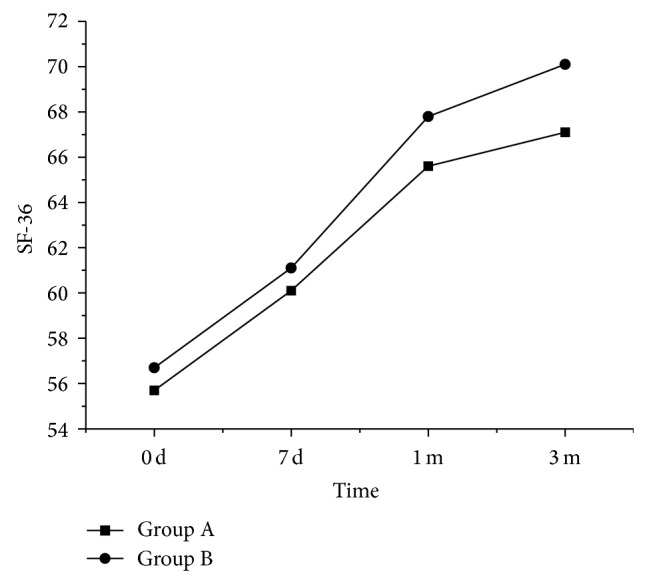
SF-36 scale changes before and after treatment in patients of groups A and B.

The quality of life of patients was similar between groups A and B before treatment, in seven days of treatment, and in one and three months of treatment (Figure 4).

The results above based on SF-36 evaluation suggested that the quality of life of patients with chronic trapezius MPS could be improved in one to three months after treatment using simply DN and DN heating therapy. The effects of the two treatments on the quality of life of patients MPS were similar in seven days, one month, and three months after treatment.

The PPT values showed that the pain in patients of group A was relieved after seven days, one month, and three months after treatment than before treatment. But the PPT values were similar in seven days, one month, and three months after treatment. The pain in patients of group B was relieved after seven days, one month, and three months after treatment more than before treatment, and after one month, and three months than after seven days, but not significantly different in one and three months after treatment (Table 4).

**Figure 5:**
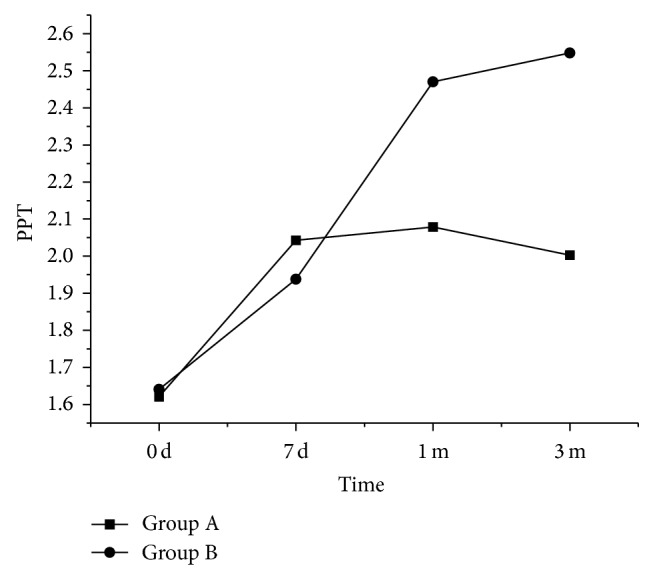
PPT values in patients of group A and group B before and after treatment.

The degree of pain in patients was similar between groups A and B before treatment and in seven days of treatment. However, the pain in patients of group B was lighter than that in group A in one and three months of treatment (Figure 5).

## 4. Discussion

Hot pack therapy can alleviate pain in patients with MPS [14] and improve the range of motion [15]. Basic study has demonstrated that the pathophysiological basis of MPS includes the formation of taut band and local hypoxia and ischemia [16], while heating can increase local blood flow and oxygen supply to the local tissues, local blood flow can quickly be restored to normal condition after heat source is removed, and the increase in oxygen supply can last for a long time [17]. Hot pack therapy is an effective treatment for MPS, but its effects are not as good as the combination therapy [18], and thus the MPS is rarely treated with hot pack therapy alone clinically.

DN is one of the methods used for treating MPS. Vulfsons et al. [19] considered that DN is effective in the treatment of myofascial trigger point. Kietrys et al. [20], through a systematic review and meta-analysis, suggested that DN is more effective in the treatment of MPS in upper extremities than conventional physical therapy, and its evidence-based medicine level achieves grade A.

In this study, the clinical observations confirmed that the effect of DN heating therapy on MPS was better than DN therapy alone.

The DN heating therapy is innovative. Traditional Chinese acupuncture can also be performed under the heating condition usually by ignited moxa stick attached at the needle tail, which is effective in the treatment of MPS [21] but different from the treatment here. The heat from ignited moxa stick at the needle tail can mainly radiate the superficial parts of the skin and subcutaneous tissue but is difficult to reach deep tissue through heat transfer. It was because the stainless steel needles used for the clinical treatment of MPS are usually thin in diameter of 0.25–0.5 mm [22–25] and the thermal conductivity of stainless steel is relatively low with about 80 W/mK so that the heat transferred from the tail into the human body is quickly taken away at the superficial tissue and the temperature at the needle tip does not change significantly before and after heating [26]. In this study, the needle was made of a variety of metals including 85% silver with thermal conductivity of 429 W/mK and smaller amounts of other metals such as copper, chromium, and nickel, with thermal conductivity higher than stainless steel. In addition, the needle diameter was 0.9 mm and could quickly transfer heat from the needle tail to the needle tip. In spite of a large amount of heat absorbed by superficial tissue, some heat was still transmitted to the needle tip. Previous studies have demonstrated that heating the needle tail can increase temperature at the tip of the needle mainly made of silver [27, 28]. Therefore, the DN heating therapy studied could heat deep tissues, especially taut bands that the needle tip could reach, thereby improving the ischemic-hypoxic condition of lesions and clinical efficacy.

The heating device used in this study could achieve stable heating and precise temperature adjustment. After reaching the target value in one-two min, the temperature detected by temperature-measuring device through feedback of bamboo-style heating apparatus was kept constant, which met the requirements for treatment and ensured that patients were not damaged by high temperature.

### 4.1. Changes in Pain Intensity

#### 4.1.1. VAS and PPT

The VAS method can be affected by many subjective factors such as mental stress [29], but it is simple and easy to operate and understand by patients; thus it has good reliability and validity [30] and is still widely used in clinical practice.

This study based on the VAS evaluation showed that both DN only and DN heating therapy were effective for chronic and trapezius MPS, and the therapeutic effect could last for at least three months. After one and three months of treatment, DN heating treatment had better effects on MPS than on the DN treatment.

The quantitative measurement of PPT was first proposed by Libman [31] in 1934. Hereafter, PPT determination has been increasingly used in clinical treatment. Studies have confirmed high reliability of PPT measurement in clinical treatment [32, 33] and for the healthy population [34]. The PPT has been widely applied for evaluation of MPS and various skeletal muscle diseases [35] and evidenced for its good reliability and validity in the evaluation of MPS [36].

Current researches on PPT mostly focus on the trapezius muscle and infraspinatus. It was reported that the PPT of trapezius muscle is 3.7 kg/cm^2^ in normal female adult [35], 2.3 kg/cm² in female telephone operator [37], and 3.1 kg/cm² in normal male adult [38]. Kwon et al. [39] detected 20 female patients with MPS and found the PPF averaged 5.4 kg/cm². This study revealed that the PPT in patients with MPS was 1.6 kg/cm² before treatment, 2.0 kg/cm² after DN treatment, and 2.0 kg/cm² after DN heating treatment. The results were consistent with those found by Lee et al. [37] but different from those reported by Lee et al. [38], which may be caused by factors such as operating skills, measurement tools, gender of patients, dominant location of trigger points, and body mass index [36].

This study based on PPT method demonstrated that both DN and DN heating treatment could treat MPS and the therapeutic effect could last at least for three months. In one and three months of treatment, the effect of DN heating treatment was better than that of DN treatment.

#### 4.1.2. Quality of Life

SF-36 is the most widely used tool for physical and mental health assessment and is increasingly applied to evaluate patients with pain [40, 41]. Its evaluation is based on the answers of patients to eight health-related questions and one question about changes in their physical condition in the past year [42]. Its reliability and validity for evaluation of chronic pain disorders have been confirmed [29, 43]. The eight health-related questions are (1) limitations on physical activities because of health problems, (2) limitations on social activities because of physical or emotional problems, (3) limitations on usual role activities because of physical health problems, (4) bodily pain, (5) general mental health (psychological distress and wellbeing), (6) limitations on usual role activities because of emotional problems, (7) vitality (energy and fatigue), and (8) general health perceptions [44]. The Chinese version of the SF-36 rating scale was used here. SF-36 score ranged from 0 to 100, with 0 suggesting poor physical condition and 100 indicating the best physical condition.

The SF-36-based evaluation here showed that both methods were effective for chronic trapezius MPS, and their effects were not different, which was different from the results derived from VAS and PPT evaluation. It was probably because the DN treatment including TN heating therapy primarily focused on the pain in patients. The VAS and PPT were used to assess the degree of pain and more sensitive to the degree of pain, while the SF-36 is mainly for evaluation of the overall physical condition of patients that included pain and others. Although pain might be included in the other items, it did not account for a high proportion in the SF-36 evaluation and hence SF-36 evaluation was less sensitive to pain changes than VAS and PPT.

The limitations of the DN heating treatment were associated with the large diameter of needles. Although lidocaine was used for surface anesthesia before acupuncture, patients still could feel the obvious pain during the treatment because the needle was injected into a relatively deep position. Fortunately, the pain was within the acceptable range of most patients, occurred mainly in the process that the needle was inserted, and was not obvious when the needle remained in the body and heated, which was evidenced by no sample loss during the treatment due to pain caused by inserting needle.

## 5. Conclusions

Dry needle treatment of chronic trapezius muscle myofascial pain syndrome is effective. And our research showed that doing needle treatment conduction through the needle tail heating was safe, evaluation methods of VAS and PPT confirmed that heating dry needle in improving the degree of pain had a better long-term curative effect than just dry needle therapy.

## Figures and Tables

**Table 1:** Demographic characteristics of study participants.

Parameter	Group A	Group B	*P* value
*n*	28	29	
Sex (male/female)	11/17	10/19	0.460
Age (years)	44.07 ± 11.71	41.83 ± 11.72	0.473
Disease duration (mo)	53.04 ± 36.30	44.07 ± 32.90	0.332
BMI	23.01 ± 2.64	2.80 ± 2.90	0.775
Location trigger point (dominant/nondominant)	17/11	15/14	0.339

BMI: body mass index.

**Table 2:** Visual analogue scale (mean ± SD).

VAS						
Parameter	0 d	7 d	1 m	3 m	p	
A group	6.5 ± 1.2	5.7 ± 1.3	5.5 ± 1.3	5.8 ± 1.2	p1 = 0.014	p2 = 0.005
					p3 = 0.034	p4 = 0.708
					p5 = 0.723	p6 = 0.466

B group	6.4 ± 1.1	5.1 ± 1.7	4.6 ± 1.9	4.7 ± 2.1	p1 = 0.003	p2 = 0.000
					p3 = 0.000	p4 = 0.342
					p5 = 0.454	p6 = 0.840

*P*	0.827	0.15	0.043	0.024		

p1: comparison between 0 and seven days of treatment; p2: comparison between 0 and one month of treatment; p3: comparison between 0 and three months of treatment; p4: comparison between seven days and one month of treatment; p5: comparison between seven days and one month of treatment; p6: comparison between one month and three months of treatment.

**Table 3:** SF-36 evaluation before and after treatment in patients of groups A and B (mean ± SD).

SF-36						
Parameter	0 d	7 d	1 m	3 m	p	
A group	55.7 ± 11.8	60.1 ± 13.0	65.6 ± 12.8	67.1 ± 13.1	p1 = 0.198	p2 = 0.004
					p3 = 0.001	p4 = 0.108
					p5 = 0.042	p6 = 0.667

B group	56.7 ± 11.8	61.1 ± 11.3	67.8 ± 10.0	70.1 ± 10.7	p1 = 0.128	p2 = 0.000
					p3 = 0.000	p4 = 0.023
					p5 = 0.002	p6 = 0.424

*P*	0.757	0.758	0.481	0.348		

p1: comparison between 0 and seven days of treatment; p2: comparison between 0 and one month of treatment; p3: comparison between 0 and three months of treatment; p4: comparison between seven days and one month of treatment; p5: comparison between seven days and one month of treatment; p6: comparison between one month and three months of treatment.

**Table 4:** Pressure pain threshold (mean ± SD).

PPT kg/cm^2^						
Parameter	0 d	7 d	1 m	3 m	p	
A group	1.62 ± 0.51	2.04 ± 0.75	2.07 ± 0.76	2.00 ± 0.70	p1 = 0.024	p2 = 0.015
					p3 = 0.040	p4 = 0.847
					p5 = 0.832	p6 = 0.685

B group	1.64 ± 0.31	1.94 ± 0.48	2.47 ± 0.63	2.55 ± 0.68	p1 = 0.018	p2 = 0.000
					p3 = 0.000	p4 = 0.016
					p5 = 0.012	p6 = 0.911

*P*	0.859	0.532	0.039	0.004		

p1: comparison between 0 and 7 days of treatment; p2: comparison between 0 and 1 month of treatment; p3: comparison between 0 and 3 months of treatment; p4: comparison between 7 days and 1 month of treatment; p5: comparison between 7 days and 1 month of treatment; p6: comparison between 1 month and 3 months of treatment.
